# Extraskeletal Ewing’s Sarcoma of Neck in a Child- A Case Report

**Published:** 2019-05

**Authors:** Mohammed-Humaam Ansari, Atishkumar-Balajirao Gujrathi, Vijayalaxmi Ambulgekar

**Affiliations:** 1 *Department of Otorhinolaryngology, Dr. Shankarrao Chavan Government Medical College, Nanded, Maharashtra, India. *

**Keywords:** Child, EES, Extraskeletal, Ewing’s sarcoma, Neck

## Abstract

**Introduction:**

Ewing's sarcoma (ES) is an extremely rare bone malignancy observed in otorhinolaryngeal practice. In otorhinolaryngology, it sometimes involves the facial bones and cervical vertebrae. In children, ES is the second most common primary malignancy of bone after osteosarcoma. Extraskeletal Ewing’s sarcoma (EES) is an extremely rare malignancy of mesenchymal cell origin. The EES is observed in the trunk and lower extremities. The neck is an extremely rare primary site for EES.

**Case Report:**

A 3-year-old female child referred with a rapidly enlarging right-sided neck swelling since 2 months ago. Physical examination and preoperative investigations suggested the diagnosis of a carotid body tumor and histopathological findings were similar. However, the results of immunohistochemistry revealed a confirmatory diagnosis of ES.

**Conclusion:**

Hereby, we presented a case of EES of the neck in a child, which was completely misdiagnosed preoperatively due to its rare incidence. The incidence of EES in the head and neck region can be successfully managed with radical excision and radiotherapy.

## Introduction

Ewing's sarcoma (ES) is a locally aggressive bone malignancy that is observed more commonly in males, compared to females in the first three decades of life (1). The ES is most frequently detected in long bones. The tumor is rarely noticed in the head and neck region involving the facial bones and cervical vertebrae. The localized ES of orbit, retropharynx, and nose have been reported (2-4). Primary ES of the head and neck region is very rare, including only 1% to 4% of all cases with ES (5). Many researchers believe that ES of the head and neck region has a good prognosis, compared to ES in other parts of the body (2-5). In children, ES is the second most common primary malignancy of bone after osteosarcoma. Extraskeletal Ewing's sarcoma (EES) is a round cell malignancy of mesenchymal cell origin. The ESS is very rare in otorhinolaryngeal practice. It is a soft tissue tumor that is morphologically similar to the ES of bone; however, it has a higher propensity of distant metastasis. Tefft et al. described four cases of paravertebral soft tissue tumors that were histologically similar to ES (6). Later, Angervall and Enzinger described 39 cases of soft tissue paravertebral tumors with similar morphological characteristics of ES of bone (7). Chao et al. reported that out of 118 patients with ESS, only 5 cases were identified with ESS located in head and neck (8). 

## Case Report

A 3-year-old female child was referred to our Head and Neck Department complaining about right-sided neck swelling since 2 months ago. The case had no pain in swelling, fever, trauma, dysphagia, and dyspnoea. Physical examination revealed a palpable mass of 4×3 cm in the right side carotid triangle that was firmly consistent, as well as nontender and nonfluctuant in nature. There was no evidence indicating the movement of swelling on deglutition. Overlying skin was reported to be normal. In addition, there were no signs of inflammation. Cervical lymph nodes were normal and systemic examination was unremarkable.

**Applied Examinations**

No abnormality was observed in the results of haemogram, chest X-ray, and blood chemistries. Computed tomography scan showed a well-circumscribed, isodense, and nonenhancing soft tissue mass with a size of 46×40 mm along the carotid space on right side. Bilateral thyroid gland appeared to be normal in size, shape, and echogenicity. Bilateral submandibular glands, parotid glands, and carotid arteries appeared normal. Laryngeal and parapharyngeal structures were also normal. The diagnosis was carotid body tumor and fine-needle aspiration cytology was not performed. 

**Procedure:**

The patient was subjected to excision of the cervical mass. She underwent the surgery under general anesthesia with orotracheal intubation. A horizontal incision was carried out on the right side of the neck from the posterior border of right sternomastoid to the midline. The subplatysmal flap was elevated. 

**Fig 1:**
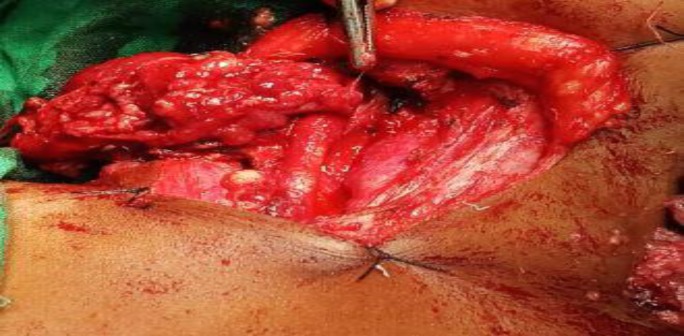
Swelling intraoperative encasing right common carotid artery

**Fig 2:**
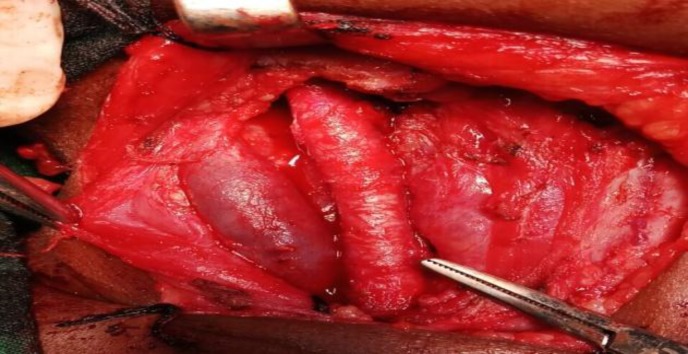
Right internal carotid artery and right internal jugular vein intraoperative preservation

After dissecting the fibers of strap muscles, the swelling was discovered encasing the right common carotid artery from which it was carefully dissected and separated (Fig.1). The mass was completely removed as the adjacent structures were not infiltrated. The right-sided common carotid artery and internal jugular vein were preserved (Fig.2). Then it was followed by an uneventful recovery. The clinical follow-up of the patient has demonstrated no recurrence so far.

**Gross Examination:**

The excised mass was greyish white, 4x3x1 cm in size, as well as soft and cystic. In the incised section, the cystic cavity was noted at one end, along with papillary projections.

**Histological examination:**

Histopathological examination showed that the tumor consisted of large round to oval cells having vesicular nuclei with prominent nucleoli and scanty eosinophilic cytoplasm. The tumor cells were observed in zellballen pattern. Intervening fibrocollagenous stroma was infiltrated by mononuclear cells and congested blood vessels. Microscopic features were also suggestive of carotid body tumor.

**Fig 3:**
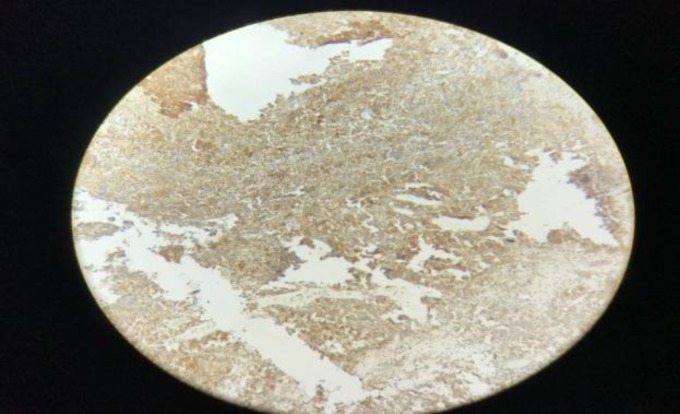
Positive immunohistochemical staining with CD99

**Fig 4:**
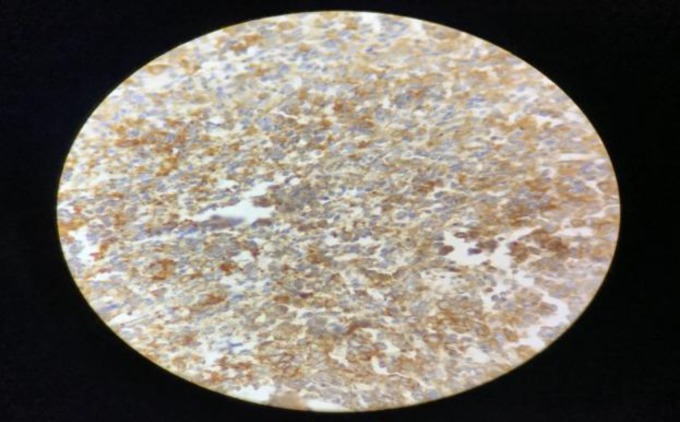
Positive immunohistochemical staining with CD99

**Fig5:**
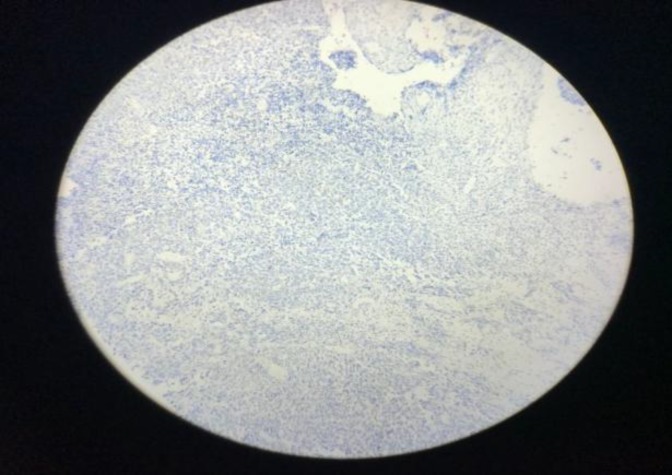
Negative immunohistochemical staining with S100

**Fig 6:**
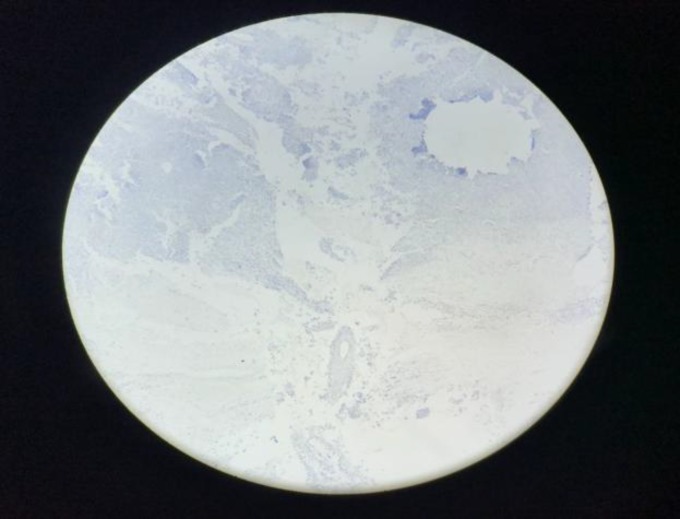
Negative immunohistochemical staining with desmin

**Fig 7:**
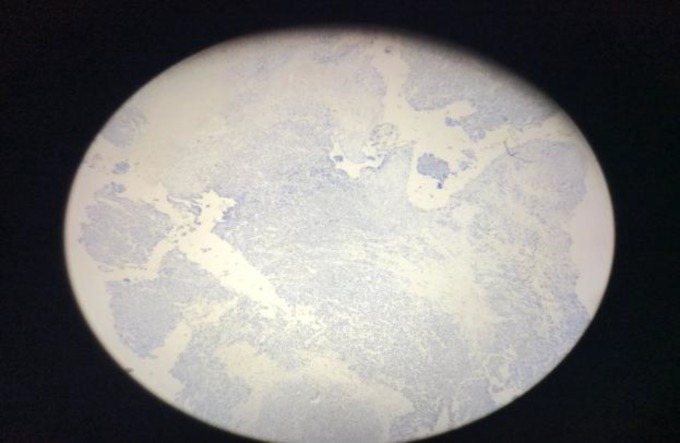
Negative immunohistochemical staining with synaptophysin

**Fig 8:**
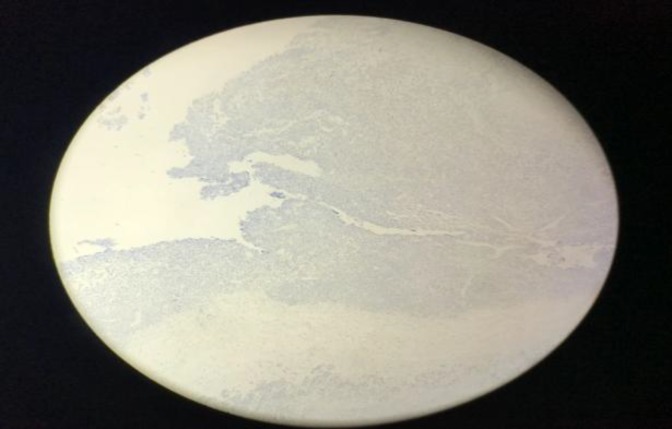
Negative immunohistochemical staining with transducin-like enhancer of split-1

The results of immunohistochemistry showed immunoreactivity to Mi-2 protein (CD99), vimentin with paranuclear immunopositivity for pancytokeratin. The tumor is immunonegative for leukocyte common antigen (LCA/CD45), S100 protein, desmin, myogenin, synaptophysin, cluster of differentiation 31, ETS-related gene, and transducin-like enhancer of split-1. Immunohistochemical findings were confirmatory for ES (Fig. 3-8). The results of a positron emission tomography scan of the whole body were normal. Then the patient was treated by means of radiotherapy. 

## Discussion

The ES comprises about 4% to 6% of all primary bone malignancies (1,5). The ES arises in the marrow cavity in the epiphyses of long bones. The ES of the head and neck region is extremely rare, including only 1% to 4% of the cases (1,5). It rarely occurs after the third decade of life and is most common in the second decade of life that is frequently observed in males in comparison to females (1,5). 

The EES is an infrequent malignancy of mesenchymal cell origin. The EES generally occurs in the soft tissues of the trunk and lower extremities. The head and neck region is an uncommon primary site for EES. Chao et al. described only 5 out of 118 cases of EES localized in the head and neck region (8). 

The individuals within the age range of 10-30 years are predominantly affected (2,9). Most patients have a painless mass, which is indicative of rapid growth. One-third of the cases presented distant metastasis at the time of diagnosis. The histological image of EES is a round cell with scanty cytoplasm. For the first time, Angervall and Enzinger described the features and behavior of EES (7). The ES expresses CD99 on their cell membranes and therefore antibody staining for CD99 confirms the diagnosis. Rhabdomyosarcoma and lymphoblastic lymphoma can be ruled out by negative staining for LCA, myoglobulin, actin, cluster of differentiation 30, and myosin. Neuroblastoma can be excluded by negative staining for neurofilament, neuron-specific enolase, and S100 protein. Diagnosis of our patient was carried out after extensive immunohistochemical staining.

The management of Ewing's sarcoma consists of surgical removal of the tumor followed by radiotherapy and chemotherapy. Radiotherapy exclusively has high chances of local recurrences; consequently, a radical surgical removal is necessary to reduce the chances of recurrence. The neck tumors have a better survival ratio in comparison to other anatomic locations (5). Five-year survival rates of EES are reported between 38% and 67%. The EES has a poor prognosis; nonetheless, the surgical excision with chemotherapy and radiotherapy improves the chances of survival (9,10). 

## Conclusion

Despite the fact that ES of the head and neck region is very rare, it must be considered as an important differential diagnosis. Preoperative investigations require a high index of suspicion. It is recommended to perform a complete surgical resection. Even though ES has a poor prognosis, it was concluded that ES of the head and neck region can be successfully treated with adequate excision and radiotherapy.
